# Supplementary material for: Genetic Risk Variants for Class Switching Recombination Defects in Ataxia-Telangiectasia Patients
Source: J Clin Immunol. 2021 Oct 10;42(1):72–84. doi: 10.1007/s10875-021-01147-8 (PMC8821084; doi:10.1007/s10875-021-01147-8)
Supplement: Supplementary file 1 — Supplementary file1 (766 21 KB) [file 10875_2021_1147_MOESM1_ESM.docx]

**Genetic Risk Variants for Class Switching Recombination Defects in Ataxia-Telangiectasia Patients**

Parisa Amirifar^1,2^, Mahya Mehrmohamadi^3^, Mohammad Reza Ranjouri^2^, Seyed Mohammad Akrami^1^, Nima Rezaei^2,4^, Ali Saberi^5^, [Reza Yazdani](http://www.sciencedirect.com/science/article/pii/S0091674917314367?via%3Dihub#!)^2^^,4^, Hassan Abolhassani^6,7,2*^, [Asghar Aghamohammadi](http://www.sciencedirect.com/science/article/pii/S0091674917314367?via%3Dihub#!)^2*^

*1. Department of Medical Genetics, School of Medicine, Tehran University of medical sciences, Tehran, Iran.*

*2. Research Center for Immunodeficiencies, Pediatrics Center of Excellence, Children's Medical Center, Tehran University of Medical Science, Tehran, Iran.*

*3. Department of Biotechnology, College of Science, University of Tehran, Tehran, Iran*

*4. Primary Immunodeficiency Diseases Network (PIDNet), Universal Scientific Education and Research Network (USERN), Tehran, Iran.*

*5. Department of Computer Engineering, Sharif University of Technology, Tehran, Iran.*

*6. Division of Clinical Immunology, Department of Biosciences and Nutrition, Karolinska Institute, Stockholm, Sweden.*

*7. Division of Clinical Immunology, Department of Laboratory Medicine, Karolinska Institute at Karolinska University Hospital Huddinge, Stockholm, Sweden.*

*** Corresponding author:** Hassan Abolhassani, MD, MPH, PhD.

**Address:** Division of Clinical Immunology, Department of Biosciences and Nutrition, NEO, Blickagangen 16, Karolinska Institute, Stockholm, SE-14157, Sweden

**Tel:** +46 8 5248 1117

**Email:** [hassan.abolhassani@ki.se](mailto:hassan.abolhassani@ki.se)

*** Corresponding author:** [Asghar Aghamohammadi](http://www.sciencedirect.com/science/article/pii/S0091674917314367?via%3Dihub#!), MD, PhD.

**Address:** Children’s Medical Center Hospital, 62 Qarib St., Keshavarz Blvd., Tehran 14194, Iran.

**Tel:** + 98 21 6642 8998

**E-mail:** [aghamohammadi@sina.tums.ac.ir](mailto:aghamohammadi@sina.tums.ac.ir)

**Running title:** Modifier genes in AT-CSR defect

**Abstract**

***Background:*** Ataxia-telangiectasia (A-T) is a rare autosomal recessive disorder caused by mutations in the *ataxia telangiectasia mutated* (*ATM*) gene. A-T patients manifest considerable variability in clinical and immunological features, suggesting the presence of genetic modifying factors. A striking heterogeneity has been observed in class switching recombination (CSR) in A-T patients which cannot be explained by the severity of ATM mutations.

***Methods:*** To investigate the cause of variable CSR in A-T patients, we applied Whole-Exome Sequencing (WES) in 20 A-T patients consisting of 10 cases with CSR-defect (CSR-D) and 10 controls with normal CSR (CSR-N). Comparative analyses on modifier variants found in the exomes of these two groups of patients were performed.

***Results:*** For the first time, we identified some variants in the exomes of the CSR-D group that were significantly associated with antigen processing and presentation pathway. Moreover, in this group of patients, the variants in four genes involved in DNA double-strand breaks (DSB) repair signaling, in particular, *XRCC3* were observed, suggesting an association with CSR defect.

***Conclusion:*** Additional impact of certain variants, along with *ATM* mutations, may explain the heterogeneity in CSR defect phenotype among A-T patients. It can be concluded that genetic modulators play an important role in the course of A-T disease and its clinical severity.

**Keywords:** Primary immunodeficiency, Inborn errors of immunity, Ataxia-telangiectasia (A-T), ATM, Class switching recombination (CSR), DNA repair, Modifier genes, Whole-exome sequencing.

**Article summary line:** This study presents a new approach toward the identification of genetic modifiers involved in the severe presentation of ataxia-telangiectasia associated with disturbed class switching recombination, suggesting involved variants in antigen presentation process and DNA repair pathways.

**Introduction**

Ataxia-telangiectasia (A-T), also known as Louis-Bar syndrome (OMIM #208900), is an autosomal recessive disorder caused by mutations in the *ataxia telangiectasia mutated* (*ATM*) gene encoding a serine/threonine-protein kinase (ATM) [[1](#_ENREF_1), [2](#_ENREF_2)]. A-T patients exhibit a broad range of clinical manifestations, including progressive cerebellar ataxia, oculocutaneous telangiectasia, variable immunodeficiency, radiosensitivity and susceptibility to malignancies [[3](#_ENREF_3), [4](#_ENREF_4)]. Other phenotypes such as infections, pulmonary diseases, insulin-resistant diabetes, growth failure, gonadal atrophy, cutaneous abnormality, metabolic and cardiovascular disease have also been reported in these patients [[5](#_ENREF_5), [3](#_ENREF_3), [6-9](#_ENREF_6)]. The ATM protein plays a major role in DNA double-strand breaks (DSB) repair, cell cycle regulation and genomic stability [[10](#_ENREF_10), [11](#_ENREF_11)]. Furthermore, ATM plays important role in B and T cell development (particularly in antigen receptor rearrangement) and class switching recombination (CSR) in mature B cells [[12](#_ENREF_12), [13](#_ENREF_13)].

Overall, A-T patients manifest significantly variable clinical and immunological features without genotype-phenotype correlation, involving modifying factors. Based on serum immunoglobulins (Ig) profile, patients with A-T could be assigned to one of the following subgroups: normal Ig level, IgA deficiency, hypogammaglobulinemia, and hyper IgM (HIgM) phenotype known as Ig CSR defect (CSR-D) [[14-17](#_ENREF_14)]. The most frequent immunodeficiencies in A-T individuals are related to IgG2 and IgA deficiency [[15](#_ENREF_15), [18](#_ENREF_18)]. On the other hand, a minority of A-T patients present HIgM phenotype, who manifest low switched Igs (IgG, IgA, and IgE) with normal or increased IgM [[19](#_ENREF_19), [20](#_ENREF_20)]. In our previous study performed in 2017, we showed that about 20% of A-T patients show Ig CSR-D [[21](#_ENREF_21)]. Generally, A-T patients with HIgM experience a more severe course of the disease leading to a lower quality of life and shorter survival [[22](#_ENREF_22)].

Some previous studies have confirmed the role of ATM in the CSR mechanism [[12](#_ENREF_12), [13](#_ENREF_13), [23](#_ENREF_23)]; however, the causative pathogenesis of CSR-D phenotype compared to patients with normal CSR (CSR-N) in A-T patients remains unclear. T cell abnormality and absence of germinal center activation due to cellular defect has been proposed, which was failed when compared between CSR-D and CSR-N A-T patients [[24](#_ENREF_24)]. It has been hypothesized that the type or the location of *ATM* mutations may be the cause of CSR defect in some A-T patients but the observation of different CSR phenotypes in patients with the same mutations falsified this anticipation [[16](#_ENREF_16)]. On the other hand, other genetic factors could be involved in CSR-D in A-T patients. Nevertheless, no data is available to determine the molecular level on the modification of ATM activity by other signaling proteins. Towards a better understanding of the phenomenon of CSR, we classified our A-T patients into two groups based on CSR status and compared the genotype of the two groups by Whole-Exome Sequencing (WES). In this study, for the first time, we investigated variations in genes other than *ATM* that might be attributed to CSR-D phenotype in A-T patients. The majority of the variants we found have known roles in the CSR mechanism, suggesting them as potential candidates for further investigation in the future.

**Materials and methods**

***Patients***

In this study, we recruited 20 unrelated A-T patients (11 females and 9 males) from the Iranian Immunodeficiency Registry Center at Children's Medical Center hospital in Tehran, Iran [[25](#_ENREF_25)]. Diagnosis of A-T patients was performed according to the European Society for Immunodeficiency (ESID) guideline [[26](#_ENREF_26)], including ataxia and at least two of the following: oculocutaneous telangiectasia, elevated alpha-fetoprotein (AFP), lymphocyte A-T karyotype with translocation chromosome 7:14 and cerebellar hypoplasia on magnetic resonance imaging (MRI).

***Classification of patients based on CSR***

Based on serum Ig levels, A-T patients studied were classified into 2 groups: CSR-D and CSR-N. A-T patients who had a normal serum IgA, IgG, IgM and IgE were classified as CSR-N. On the other hand, A-T patients with decreased IgG, IgA, and IgE levels (at least 2SD below normal for age), but normal to increased IgM and/or D (at least 2SD above normal for age) levels were classified as CSR-D. A-T patients with other types of antibody deficiency (e.g. IgA/IgG subclass deficiencies) were not included since they present residual CSR function. The amplification of Sμ-Sα fragments from genomic DNA by nested PCR strategy and *in vitro* sCD40L+rIL-4-induced B-cell proliferation by cell culture was performed to evaluate the capabilities of CSR toward IgA and IgE production in all patients, respectively, as described in our previous study [[22](#_ENREF_22)]. Of note, each A-T individual’s samples have run on a separated gel to take an overall quantitative measure (%), therefore the exposure of gels was not the measured values and does not have any impact on this quantitative outcome; all gels counted also in overexposure and triplicate experiments to avoid selection bias/sample bias and reported in as groups classified (CSR-D and CSR-N).

***Whole-exome sequencing and bioinformatic analysis***

The patient's peripheral blood was obtained, and DNA was extracted using the salting-out method, as previously described [[27](#_ENREF_27)]. For all patients WES was performed to detect single nucleotide variants, insertion/deletions and copy number variations using a pipeline described previously [[28](#_ENREF_28), [29](#_ENREF_29)]. Candidate variants were evaluated by the Combined Annotation Dependent Depletion (CADD) algorithm and an individual gene cutoff given by using the Mutation Significance Cutoff (MSC) was considered for impact predictions [[30](#_ENREF_30)]. The Gene Damage Index (GDI) server and the Human Gene Connectome (HGC) were used to making a combined effect prediction [[30](#_ENREF_30)]. The pathogenicity of all disease attributable gene variants was re-evaluated using the updated guideline for interpretation of molecular sequencing by the American College of Medical Genetics and Genomics criteria (ACMG) [[31](#_ENREF_31), [32](#_ENREF_32)].

***Case-control association analysis***

We used Genome-Wide Analysis Toolkit (GATK) Haplotypecaller for joint variant calling on all 20 samples. We then performed a case-control association analysis on the variant-allele frequencies (AFs) using the SnpSift CaseControl tool taking into account four different genetic testing models including trend, allele count, dominant and recessive models [[33](#_ENREF_33)]. The statistical tests used were the Cochran–Armitage test for trends and Fisher’s exact test for the allele count, dominant, and recessive models. The Fisher’s exact test between case and control was also repeated at the gene-level by aggregating allele counts across all variants annotated to the same gene in the genome. Cochran-Armitage and Fisher’s exact statistical tests were performed to identify statistically significant variants between two groups of A-T patients. A q-value (using Bonferroni correction) of less than 0.05 was considered statistically significant. Next, functional annotation and pathway enrichment analysis for significant genes/variants identified from all methods were performed by “EnrichR” (comprehensive gene set enrichment analysis extracting resources from Gene Ontology [GO], Kyoto Encyclopedia of Genes and Genomes [KEGG]) and “DAVID” (Database for Annotation, Visualization and Integrated Discovery extracting Protein ANalysis THrough Evolutionary Relationships [PANTHER] data), which is a comprehensive gene set enrichment analysis database (20, 21).

***Statistical analysis***

Statistical analysis was conducted by SPSS software package version 21.0 (SPSS Inc., Chicago, IL, USA). Median and interquartile range (IQR) were calculated and compared for demographic data and laboratory findings of A-T patients using the Mann-Whitney U test. To analyze the categorical variables from the frequency table, the Chi-square test or Fisher's exact test was performed.

**Results**

***Clinical characterization***

Based on patients’ immunologic profiles, we considered 20 unrelated A-T patients [10 A-T patients with normal CSR (CSR-N) and 10 A-T patients with CSR defect (CSR-D)] with the median interquartile range (IQR) age of 5.0 (4.2-7.7) years old at the time of diagnosis. All the patients suffered from ataxia and telangiectasia. Other presentations of our patients were recurrent infections (60%) predominated by respiratory infections (50%), followed by diarrhea (35%), dermatologic manifestations (30%), hepatosplenomegaly (35%), and autoimmunity (15%). We found that the frequency of total infections and respiratory infections in A-T patients with CSR-D were significantly higher than in the CSR-N group (*p*=0.020 and *p*=0.025, respectively). Increased serum level of AFP was seen in all patients**,** but there was no significant difference between the serum AFP concentrations of the two subgroups. The main demographic, clinical and laboratory characteristics of the patients are provided in **Table 1**. In addition, the distribution of immunoglobulins for each patient in both groups is shown in **Figure S1**.

To further ensure the accuracy of patient classification, cell culture and nested-PCR were used to confirm the capabilities of CSR toward IgE and IgA in all A-T patients, respectively, as described in our previous study [[22](#_ENREF_22)]. As expected, IgE production (**Figure S2**) was perturbed in patients with CSR-D, but not in the CSR-N group. Moreover, pooled data of the CSR-D group prove that IgA memory B-cells or plasmablasts decreased in these patients and indicate another line of evidence toward the possibility of CSR defects compared to other AT patients with IgA memory B-cells or plasmablasts which the machinery of CSR and DNA repair must function correctly to produce these B cell subsets (**Figure S3**).

***Genetic characterization***

Pathogenic mutations in the *ATM* gene were detected in all 20 patients using WES (null or deleterious mutations in 90% of CSR-D and 80% of CSR-N). So, CSR-N patients did not have significantly higher missense/hylomorphic mutations compared to the CSR-D group. We detected a homozygous mutation in the *ATM* gene for 16 out of 20 patients (80%), while a compound heterozygous mutation was found in four patients (20%, 2 CSR-D and 2 CSR-N patients), as described in **Table S1**.

We next performed a detailed analysis of additional variants found in other genes except for *ATM*. For identifying possible mutations associated with the CSR defect, we compared variant distributions among the two groups of CSR-D and CSR-N patients using SNPsift [[33](#_ENREF_33)]. We obtained 1645 variants (1074 unique genes) that were statistically significantly different between the two groups CSR-D and CSR-N) q-values <0.05). To understand the potential functional consequences of the 1645 variants, enrichment analysis was performed (**Figure 1**).

All subgroups of the three gene ontology (GO) categories (biological process, cellular component, and molecular function) were assessed for enrichment. The GO cellular component enrichment revealed an association with the plasma membrane (q=0.00014) (**Table S2**). Pathway enrichment for the KEGG annotated pathways showed a significant representation of genes in the autoimmune thyroid disease pathway (q=0.0052), allograft rejection (q=0.0204), graft-versus-host disease (GVHD) (q=0.0253), phagosome (q=0.0356), and antigen processing and presenting (q=0.044) pathways (**Table S3**). Assessment of Panther protein classes revealed that our gene list is positively correlated with immunoglobulin production (q=0.0272). Finally, among Jensen disease annotations, our gene list was significantly associated with various cancers, including carcinoma, kidney, liver, melanoma, breast and endometrial malignancies (**Figure 2** and **Table S4**).

Next, we investigated the potential pathogenic consequence of the variants found associated with CSR defect status in A-T patients. Among the 1645 variants, those with a minor allele frequency (MAF) > 0.05 in the Asian population from gnomAD (<https://www.gnomad.broadinstitute.org>), Greater Middle East Variome Project (http://igm.ucsd.edu/gme/) and Iranome dataset (<http://www.iranome.ir>) which is the most comprehensive catalog of genomic variations in the Iranian population to date, and also those that were not considered as damaging based on Sorting Intolerant From Tolerant (SIFT) and Combined Annotation Dependent Depletion (CADD) criteria were excluded to narrow down the list, as variants with a higher chance of being damaging and potentially related to the phenotype of interest were found. Finally, there were seven variants whose specifications are provided in **Table 2.** Among these seven genes, major histocompatibility complex II (MHC II), namely human leukocyte antigen (HLA) DR-Beta-5 (HLA-DRB5( is considered as important protective factors in antigen processing and presentation pathway, suggesting that the underlying cause of CSR-D is related to the dysfunction in processes related to antigen processing and presentation. The other 6 genes have no known functional connection to the CSR process (**Table 2**). Our results suggest a potential undiscovered association between these genes and the pathways involved with the CSR mechanism that should be further investigated in the future.

As a control, we next looked for overlapping variants in both CSR-N and CSR-D groups compared to the reference genome i.e. variants specifically found in A-T. After filtering and extraction of common variants among the individuals in each of the two groups, we obtained 2153 variants that were overlapping between common variants of CSR-N and CSR-D groups. We performed enrichment analysis to assess the potential functional consequences of these variants. The GO biological process enrichment revealed an association with the external encapsulating (q=0.0011) and extracellular structure organization (q=0.0034) (**Table S5**). The GO biological process enrichment showed association with the glycerophospholipid flippase activity (q=0.0106), alpha-1,4-glucosidase activity (q=0.0420), phosphatidylcholine flippase activity (q=0.0420), phosphatidylinositol trisphosphate phosphatase activity (q=0.0420), and transmembrane receptor protein tyrosine kinase activity (q=0.0420) (**Table S6**). The GO cellular component enrichment revealed an association with the collagen-containing extracellular matrix (q=0.0014), an integral component of the plasma membrane (q=0.0290), cytoplasmic vesicle membrane (q=0.0290), and endoplasmic reticulum lumen (q=0.0399) (**Table S7**). Pathway enrichment for the KEGG annotated pathways showed a significant representation of genes in the ECM-receptor interaction (q=0.0004) and protein digestion and absorption (q=0.0295) (**Table S8**). Comparison of these results with the pathway enrichment results performed on the non-overlapping variants between CSR-N and CSR-D patients (**Figure 2**) showed little similarity between the enriched pathways. This confirms the specificity of our approach in extracting potential candidates that might explain the phenotypic differences between the CSR-N and CSR-D subtypes.

***Variants involved in DSB repair pathway***

DSB repair pathway is an integral part of the CSR mechanism [[34](#_ENREF_34)] and A-T patients are known to have defects in this pathway. A supervised analysis also was conducted on all variants of DSB repair pathway-related genes with significantly different frequencies between the two patient groups. Four genes related to this pathway including MutL Homolog 1 (*MLH1*), X-Ray Repair Cross Complementing 3 (*XRCC3*), RAD23 Homolog B (*RAD23B*), and FA Complementation Group M (*FANCM*) were identified (Fisher’s exact q < 0.05). Three variants in *FANCM* were identified as protective alterations against CSR defect, while all variants of *RAD23B*, *XRCC3* and *MLH1* increased the risk. The list of the variants of these four genes, along with their characteristics, is provided in **Table 3.** All these variants were exonic and missense and had a MAF above 1% in our dataset. Also, according to SIFT and MutationTaster predicted annotations, these variants are tolerated and polymorphism, respectively. Among these variants, the *XRCC3* variant is the only variant found in all CSR-D patients as heterozygous or homozygous, while it was not observed in any of the CSR-N patients (**Figure 3**). Howbeit, we did not observe differences in the severity of clinical and immunological profiles between homozygous and heterozygous variants of *XRCC3* in the CSR-D group. XRCC3 is involved in the homologous recombination repair (HRR) pathway of DSB DNA repair [[35](#_ENREF_35)], suggesting a strong candidate for explaining variation in CSR mechanism among A-T patients.

Next, to assess the specificity of our results in the candidate pathway approach, we looked for DSB pathway-related variants among overlapping common variants between CSR-N and CSR-D patient groups. We observed that all of the identified variants shared in both the CSR-N and CSR-D groups were indeed common polymorphisms in the Iranian population (MAF~1), and no variants were found that could be significant in terms of frequency and function (all were synonymous). Indeed, this finding further confirms the relevance of the non-overlapping variants we found in the DSB repair pathway as described in **Table 3.**

***Mutation Accumulation Analysis***

Variant-level association analysis is limited to specific variants recurrently appearing in more than one A-T patient. To further extend the association analysis to non-recurring variants, we repeated the association analysis at the gene level. In this regard, we aggregated all variants found in each gene, calculated the total variant allele count per gene in each individual, and performed a Fisher’s exact test between the two groups of cases and controls. The results of the gene-level variant association showed significant differences (Benjamini Hochberg q-value <0.05) between CSR-D and CSR-N in 110 genes (listed in Table S5). Among these genes, Fanconi anemia Complementation Group M (*FANCM*) and Mediator of DNA damage checkpoint protein 1 (*MDC1*) may be related to CSR due to their known roles facilitating a DNA damage response leading to DNA repair [[35](#_ENREF_35)]*.* Our results suggest that mutations in *FANCM* and/or *MDC1* may explain the appearance of CSR defects in A-T patients. In addition, *HLA-DRB5*, human leukocyte antigen B (*HLA-B*), HECT Domain E3 Ubiquitin Protein Ligase 1 (*HECTD1*), Moloney Leukemia Virus 10 (*MOV10*), Kinesin Light Chain 4 (*KLC4*), PH Domain and Leucine-Rich Repeat Protein Phosphatase 1 (*PHLPP1*) and Triggering Receptor Expressed on Myeloid Cells Like 4 (*TREML4*) genes with known roles in different stages of the antigen processing and presenting pathway also appear in the list of 110 genes with significant accumulation of variants (**Table S9**).

**Discussion**

In the present study, we identified variants at loci involved in antigen processing and presentation pathways and DSB repair pathway using a case-control comparative approach between groups of A-T patients differing in class switching recombination. The presence of these variants along with ATM mutations may suggest mechanisms involved in the CSR defect phenotype observed in a subset of A-T patients.

The clinical features of A-T are complex and multi-systemic, including neurological abnormalities, oculocutaneous telangiectasia, recurrent infections, immunodeficiencies, and susceptibility to cancers [[36](#_ENREF_36)]. In this study, all A-T patients exhibited ataxia and telangiectasia as the main clinical features, but there was no significant difference between the onsets of these manifestations in the two groups. Moreover, we did not observe a significant difference between the onsets of other clinical manifestations in the two groups. Recurrent infections are the most common manifestation associated with immunodeficiency in A-T patients and a major factor for early age morbidity and mortality [[5](#_ENREF_5), [15](#_ENREF_15), [37](#_ENREF_37)]. We found significantly increased episodes of infections (especially respiratory tract infections) in the CSR-D group comparing to the CSR-N group. Since environmental factors should be considered as the main modifying factor, the female frequency was higher in previously reported cases with CSR-D compared to CSR-N [[22](#_ENREF_22), [21](#_ENREF_21), [38](#_ENREF_38)], as was observed in the current assay as well.

A-T patients show variable cellular and humoral immune abnormalities [[39](#_ENREF_39)]. During the last decade, several A-T cases have been reported in whom Ig CSR defect has been implicated [[40](#_ENREF_40), [19](#_ENREF_19), [41](#_ENREF_41), [16](#_ENREF_16)]. Some previous studies reported that about 10% of the A-T patients present with the HIgM/CSR-D phenotype [[42](#_ENREF_42), [19](#_ENREF_19)]. In contrast, our previous study showed that the frequency of CSR defect in Iranian A-T patients was higher than in other populations (21.2%) [[21](#_ENREF_21)]. Previous studies have shown that the CSR junctions in cells of A-T patients are aberrant, indicating a role for ATM in the final steps of CSR, including DNA end modification, repair, and joining [[43](#_ENREF_43), [44](#_ENREF_44)], which may suggest ATM as a player in the CSR process. However, the majority of A-T patients with *ATM* mutations do not demonstrate CSR defects. Thus, the cause of this Ig profile in A-T is not entirely understood. The current study supports the notion that A-T patients with hyper IgM level, normal T cell subsets, low ability to IgE switching with stimulation of CD40L and IL-4 and having abrogated IgA memory B-cells or plasmablasts in their periphery can be classified as CSR defects, however, other hypothetical mechanisms including selective apoptosis of IgA. IgG and IgE but not IgM would be an alternative mechanism for this phenomenon (A-T patients with Ig deficiency).

Comparing the genotype of CSR-D and CSR-N patients, in the current study we proposed a new understanding of the abovementioned immunological defect. First, we rejected the hypothesis that the type, zygosity, or the location (affected domain) of ATM mutations may be the cause of CSR-D in A-T patients [[16](#_ENREF_16)], as we observed similar mutation distributions in the two groups (**Table S1** and **Figure S4**). Moreover, evaluation of the exomes of the two groups in an unsupervised manner revealed 1645 variants with significant allelic differences between the CSR-D and CSR-N groups. These variants represented enrichment terms such as antigen processing and presentation pathway, plasma membrane, autoimmunity, allograft rejection, graft-versus-host disease, and malignancy.

We found several variants in the antigen processing pathway to be associated with the CSR phenotype under study. Antigen processing and presentation is a complex process in which many molecules and proteins are involved [[45](#_ENREF_45)]. Once a B-cell receptor (BCR) recognizes a particular antigen, proteasomes degrade the antigen, and subsequently, peptide fragments were presented at the cell surface through MHC class II molecules [[46](#_ENREF_46)]. MHC class II molecules are highly polymorphic and normally expressed only on professional antigen-presenting cells such as B cells, dendritic cells, and mononuclear phagocytes. CD4^+^ T cells specific for this antigen initiate a cascade allowing the cognate B cell activation. In particular, one of the most important interactions for humoral immune responses is the engagement of CD40 molecule on B cells to the CD40 ligand on follicular helper T cells [[46](#_ENREF_46), [47](#_ENREF_47)]. At this point, the activated B cells can either differentiate into plasmablasts or get recruited into a specialized region, called germinal centers (GCs) [[48](#_ENREF_48)]. In the GC, B cells are targeted by clonal expansion, somatic hypermutation, affinity maturation, and CSR, eventually forming antibody-secreting plasma cells [[49](#_ENREF_49), [50](#_ENREF_50)]. It has been previously suggested that antigen processing and presentation are indirectly related to the quantity and quality of Ig class switching. For instance, patients with CD40/L deficiencies, known as HIgM syndrome, display an impaired production of IgG, IgA, IgE, and normal or elevated levels of IgM [[51](#_ENREF_51)]. It would therefore not be surprising to find other genes in this pathway particularly HLA-DRB5 to play a role in the CSR mechanism.

Several studies have reported that A-T patients with CSR-D present with a more severe course of the disease leading to a lower quality of life at earlier ages and shorter survival than other A-T patients [[52](#_ENREF_52), [21](#_ENREF_21), [41](#_ENREF_41)]. Moreover, A-T is a genomic instability syndrome leading to an extremely high incidence of malignancies (10-25%) [[53-55](#_ENREF_53)]. Leukemia and lymphoma account for 85% of all malignancies in A-T patients in childhood [[56](#_ENREF_56)]. However, adults are susceptible to both lymphoid tumors and various types of solid tumors including breast, liver, gastric, and esophageal carcinomas [[57](#_ENREF_57)]. Based on our results, it seems that A-T patients with CSR-D harbor additional genomic variants mainly associated with various solid tumors such as kidney, liver, melanoma, breast and endometrial cancers.

We also identified several variants in the selected DSBs repair pathway with association with the CSR phenotype in our study. DSBs are potentially lethal lesions occurring as a result of exposure to exogenous agents such as radiation and certain chemicals [[10](#_ENREF_10)]. DSBs also occur as intermediates in various biological events, such as V(D)J recombination and efficient CSR [[58](#_ENREF_58), [59](#_ENREF_59)]. The most common pathways used to repair DSBs are non-homologous end joining (NHEJ) and homologous recombination (HR) [[60](#_ENREF_60)]. Generally, an early event during the DSB response is the activation of ATM protein, leading to rapid phosphorylation of several proteins involved in DNA repair, cell cycle checkpoint, and transcription regulation. Based on the importance of the DSB repair pathway in CSR and the defect of this pathway in A-T patients, we evaluated genes that are involved in DSB repair pathways and we observed multiple variants significantly different between the two A-T groups. Remarkably, among these variants, p.T241M variant of the *XRCC3* gene, the coding protein involved in homology-directed repair, considered as a risk factor was observed exclusively in the CSR-D group, and in every single case in this group. Indeed, NHEJ and alternative end-joining (A-EJ, using homology-directed repair) are the main pathways involved in the repair of CSR breaks. However, some findings demonstrate that although AID-induced breaks are repaired primarily in the G1 checkpoint by the NHEJ pathway, *Igh* DSBs that escape repair or have defects in NHEJ can persist into the S phase, where they are considerably resected and become substrates for homology-directed repair using microhomology in the S regions (A-EJ) [[61-63](#_ENREF_61)]. It seems that A-EJ mediated repair of *Igh* breaks that failed NHEJ-mediated CSR attempts would restore an intact *Igh* allele for the next round of AID targeting and CSR. In fact, these findings suggest that A-EJ contributes to the repair of CSR-related DSBs [[61-64](#_ENREF_61)]. The main component involved in A-EJ is usually XRCC1 to recruit LIG3, however recent studies observed XRCC1 independent microhomology-mediated A-EJ with a tight connection of PARP1 and XRCC3 [[65](#_ENREF_65), [66](#_ENREF_66)]. On the other hand, a few studies reported a role for HR in proliferation and genome stability in early B cell development [[67](#_ENREF_67), [68](#_ENREF_68)]. Caddle et al. [[67](#_ENREF_67)] have demonstrated that HR, with a major role of XRCC3, is essential for the promotion of lymphocyte differentiation or maturation. The study showed that the functions of *XRCC2*, a homolog member of the RECA/RAD51-related protein family that participates in HR, in early B cell development seem to differ from its roles in mature and activated B cells. Indeed, defective HR leads to the accumulation of AID-induced DSBs at both IGH and non-IGH loci suggesting high fidelity repair of AID-inflicted breaks is required for the B cell genome integrity [[69](#_ENREF_69), [61](#_ENREF_61)]. These were the first results to implicate HR as an important pathway with a defined role in the adaptive immune system. In fact, *XRCC2* was transcriptionally upregulated after B cell activation [[68](#_ENREF_68)]. Therefore, it seems that there is an interrelationship among B cell activation, immunoglobulin class switching and HR which is more essential in the context of NHEJ monogenic defects. In our study, we found a relationship between *XRCC3*, as another member of the RECA/RAD51-related protein family, and class switching recombination. Previous studies in cutaneous malignant melanoma and head and neck cancer have also reported this variant as a potential risk factor in DSB repair [[70](#_ENREF_70), [71](#_ENREF_71)]. Thus, it is postulated that, along with *ATM* mutations, this variant of the *XRCC3* gene plays an important role in CSR defect in A-T patients, which needs to be proved by functional studies in the future. In general, possible roles for homologous recombination, in either normal B cells development or immunodeficiency, remain controversial.

In case-control genetic studies, aggregation analysis is often used as a suitable method to identify genes associated with diseases of interest, even if variants found within them are heterogeneous in nature and position. We found that some variants of two genes involved in DSB repair, and seven genes related to antigen presentation were significantly different between our two study groups. This highlights the importance of these two mechanisms in CSR. Overall, it seems that the variants of *FANCM* and *MDC1* genes are highly important due to their effect on normal CSR mechanisms. Furthermore, our findings confirm the importance of variants of seven genes involved in antigen processing and presentation, especially *HLA-DRB5*, in normal CSR. However, it is not clear which genetic variant has larger effects on the CSR mechanism and further studies are required to elucidate this question with a higher sample size. Apparently, important causal variants in the CSR mechanism cannot be identified until functional validation assays are performed. Our study calls for further investigations of the effect of the identified variants involved in DSB response and antigen processing and presentation pathways at functional levels in A-T patients. Moreover, evaluation of other CSR defect diseases in the same pathway including MRE11 and NBN deficiencies and their severity may empower this observation in future studies.

To date, no evidence is available at the molecular level on potential modification of ATM activity by other signaling proteins and interaction partners of the ATM protein are not completely recognized [[72](#_ENREF_72)]. A comprehensive understanding of this field is required for characterizing the pathogenesis of A-T and other ATM-related diseases such as cancer. Research in this area offers a new horizon to increase our knowledge regarding ATM signaling and phenotypic diversity of patients, and perhaps these findings would be helpful in the management and prognostic estimation of the disease.

**Conclusion**

Given that similar mutations in the *ATM* gene result in different clinical phenotypes, including different immunological profiles in A-T patients, additional genetic alternations are thought to play important roles in A-T disease outcomes. In the present study, the relationship between the genotype of A-T patients and the CSR defect phenotype was investigated for the first time. Our findings showed that in addition to the *ATM* gene variants, variants in genes related to this process could help explain CSR defects in A-T patients. Further research at the functional level is required to complete and confirm the findings conclusively.

**Funding**

The authors declare that they received no financial support for the research.

**Conflict of Interest**

The authors declare that they have no conflict of interest.

**Acknowledgments**

The authors thank the Pardis Gene Technology Inc. for giving us access to computing servers. This research was supported by the Tehran University of Medical Sciences (grant no. 40601).

**Ethics declarations**

All the experiments were performed according to the guideline provided by the Ethical Committee at the Tehran University of Medical Sciences. Written Informed consent for this study was obtained from the patients and/or their parents.

**Data availability statement**

The raw data supporting the conclusions of this article will be made available by the authors, without undue reservation, to any qualified researcher.

**Authors’ Contributions**

(1) The conception and design of the study.

(2) Acquisition of data.

(3) Analysis and interpretation of data.

(4) Drafting the article.

(5) Revising it critically for important intellectual content.

(6) Final approval of the version to be submitted.

(7) Agree to be accountable for all aspects of the work in ensuring that questions related to the accuracy or integrity of any part of the work are appropriately investigated and resolved.

PA (2,3,4,5,6,7), MM, MRR, SMA, NR, AS (3,5,6,7), RY (2,3,4,5,6,7), HA and AA (1,3,4,5,6,7).

**Consent to participate**

Informed consent was obtained from the parents.

**Reference**

1. Chaudhary MW, Al-Baradie RS. Ataxia-telangiectasia: future prospects. The application of clinical genetics. 2014;7:159-67. doi:10.2147/tacg.s35759.

2. Gatti RA, Berkel I, Boder E, Braedt G, Charmley P, Concannon P et al. Localization of an ataxia-telangiectasia gene to chromosome 11q22-23. Nature. 1988;336(6199):577-80. doi:10.1038/336577a0.

3. Nissenkorn A, Levy-Shraga Y, Banet-Levi Y, Lahad A, Sarouk I, Modan-Moses D. Endocrine abnormalities in ataxia telangiectasia: findings from a national cohort. Pediatric research. 2016;79(6):889-94. doi:10.1038/pr.2016.19.

4. Teive HA, Moro A, Moscovich M, Arruda WO, Munhoz RP, Raskin S et al. Ataxia-telangiectasia - A historical review and a proposal for a new designation: ATM syndrome. Journal of the neurological sciences. 2015;355(1-2):3-6. doi:10.1016/j.jns.2015.05.022.

5. Bott L, Lebreton J, Thumerelle C, Cuvellier J, Deschildre A, Sardet A. Lung disease in ataxia‐telangiectasia. Acta paediatrica. 2007;96(7):1021-4.

6. Schalch DS, McFarlin DE, Barlow MH. An unusual form of diabetes mellitus in ataxia telangiectasia. The New England journal of medicine. 1970;282(25):1396-402. doi:10.1056/nejm197006182822503.

7. Su Y, Swift M. Mortality rates among carriers of ataxia-telangiectasia mutant alleles. Annals of internal medicine. 2000;133(10):770-8.

8. Zaki-Dizaji M, Akrami SM, Azizi G, Abolhassani H, Aghamohammadi A. Inflammation, a significant player of Ataxia-Telangiectasia pathogenesis? Inflammation research : official journal of the European Histamine Research Society [et al]. 2018;67(7):559-70. doi:10.1007/s00011-018-1142-y.

9. Zaki-Dizaji M, Akrami SM, Abolhassani H, Rezaei N, Aghamohammadi A. Ataxia telangiectasia syndrome: moonlighting ATM. Expert review of clinical immunology. 2017;13(12):1155-72. doi:10.1080/1744666x.2017.1392856.

10. Helleday T, Lo J, van Gent DC, Engelward BP. DNA double-strand break repair: from mechanistic understanding to cancer treatment. DNA repair. 2007;6(7):923-35. doi:10.1016/j.dnarep.2007.02.006.

11. Jin MH, Oh DY. ATM in DNA repair in cancer. Pharmacology & therapeutics. 2019;203:107391. doi:10.1016/j.pharmthera.2019.07.002.

12. Bakkenist CJ, Kastan MB. DNA damage activates ATM through intermolecular autophosphorylation and dimer dissociation. Nature. 2003;421(6922):499-506. doi:10.1038/nature01368.

13. Amirifar P, Ranjouri MR, Lavin M, Abolhassani H, Yazdani R, Aghamohammadi A. Ataxia-telangiectasia: epidemiology, pathogenesis, clinical phenotype, diagnosis, prognosis and management. Expert review of clinical immunology. 2020:1-13. doi:10.1080/1744666x.2020.1810570.

14. Azarsiz E, Karaca NE, Gunaydin NC, Gulez N, Ozturk C, Aksu G et al. Do elevated serum IgM levels have to be included in probable diagnosis criteria of patients with ataxia-telangiectasia? International journal of immunopathology and pharmacology. 2014;27(3):421-7. doi:10.1177/039463201402700312.

15. Nowak-Wegrzyn A, Crawford TO, Winkelstein JA, Carson KA, Lederman HM. Immunodeficiency and infections in ataxia-telangiectasia. The Journal of pediatrics. 2004;144(4):505-11.

16. Mohammadinejad P, Abolhassani H, Aghamohammadi A, Pourhamdi S, Ghosh S, Sadeghi B et al. Class switch recombination process in ataxia telangiectasia patients with elevated serum levels of IgM. J Immunoassay Immunochem. 2015;36(1):16-26. doi:10.1080/15321819.2014.891525.

17. Meyts I, Weemaes C, De Wolf-Peeters C, Proesmans M, Renard M, Uyttebroeck A et al. Unusual and severe disease course in a child with ataxia-telangiectasia. Pediatric allergy and immunology : official publication of the European Society of Pediatric Allergy and Immunology. 2003;14(4):330-3.

18. Stray-Pedersen A, Jonsson T, Heiberg A, Lindman CR, Widing E, Aaberge IS et al. The impact of an early truncating founder ATM mutation on immunoglobulins, specific antibodies and lymphocyte populations in ataxia-telangiectasia patients and their parents. Clinical and experimental immunology. 2004;137(1):179-86. doi:10.1111/j.1365-2249.2004.02492.x.

19. Noordzij JG, Wulffraat NM, Haraldsson A, Meyts I, van't Veer LJ, Hogervorst FB et al. Ataxia-telangiectasia patients presenting with hyper-IgM syndrome. Archives of disease in childhood. 2009;94(6):448-9. doi:10.1136/adc.2008.149351.

20. Amirifar P, Ranjouri MR, Yazdani R, Abolhassani H, Aghamohammadi A. Ataxia-telangiectasia: A review of clinical features and molecular pathology. Pediatric allergy and immunology : official publication of the European Society of Pediatric Allergy and Immunology. 2019;30(3):277-88. doi:10.1111/pai.13020.

21. Ghiasy S, Parvaneh L, Azizi G, Sadri G, Zaki Dizaji M, Abolhassani H et al. The clinical significance of complete class switching defect in Ataxia telangiectasia patients. Expert review of clinical immunology. 2017;13(5):499-505. doi:10.1080/1744666x.2017.1292131.

22. Amirifar P, Mozdarani H, Yazdani R, Kiaei F, Moeini Shad T, Shahkarami S et al. Effect of Class Switch Recombination Defect on the Phenotype of Ataxia-Telangiectasia Patients. Immunol Invest. 2020:1-15. doi:10.1080/08820139.2020.1723104.

23. Panchakshari RA, Zhang X, Kumar V, Du Z, Wei PC, Kao J et al. DNA double-strand break response factors influence end-joining features of IgH class switch and general translocation junctions. Proceedings of the National Academy of Sciences of the United States of America. 2018;115(4):762-7. doi:10.1073/pnas.1719988115.

24. Moeini Shad T, Yousefi B, Amirifar P, Delavari S, Rae W, Kokhaei P et al. Variable Abnormalities in T and B Cell Subsets in Ataxia Telangiectasia. J Clin Immunol. 2021;41(1):76-88. doi:10.1007/s10875-020-00881-9.

25. Abolhassani H, Kiaee F, Tavakol M, Chavoshzadeh Z, Mahdaviani SA, Momen T et al. Fourth Update on the Iranian National Registry of Primary Immunodeficiencies: Integration of Molecular Diagnosis. J Clin Immunol. 2018;38(7):816-32. doi:10.1007/s10875-018-0556-1.

26. Seidel MG, Kindle G, Gathmann B, Quinti I, Buckland M, van Montfrans J et al. The European Society for Immunodeficiencies (ESID) Registry Working Definitions for the Clinical Diagnosis of Inborn Errors of Immunity. The journal of allergy and clinical immunology In practice. 2019. doi:10.1016/j.jaip.2019.02.004.

27. Miller SA, Dykes DD, Polesky HF. A simple salting out procedure for extracting DNA from human nucleated cells. Nucleic acids research. 1988;16(3):1215. doi:10.1093/nar/16.3.1215.

28. Abolhassani H, Hammarstrom L, Cunningham-Rundles C. Current genetic landscape in common variable immune deficiency. Blood. 2020;135(9):656-67. doi:10.1182/blood.2019000929.

29. Abolhassani H, Aghamohammadi A, Fang M, Rezaei N, Jiang C, Liu X et al. Clinical implications of systematic phenotyping and exome sequencing in patients with primary antibody deficiency. Genet Med. 2019;21(1):243-51. doi:10.1038/s41436-018-0012-x.

30. Itan Y, Shang L, Boisson B, Ciancanelli MJ, Markle JG, Martinez-Barricarte R et al. The mutation significance cutoff: gene-level thresholds for variant predictions. Nat Methods. 2016;13(2):109-10. doi:10.1038/nmeth.3739.

31. Li Q, Wang K. InterVar: Clinical Interpretation of Genetic Variants by the 2015 ACMG-AMP Guidelines. Am J Hum Genet. 2017;100(2):267-80. doi:10.1016/j.ajhg.2017.01.004.

32. Richards S, Aziz N, Bale S, Bick D, Das S, Gastier-Foster J et al. Standards and guidelines for the interpretation of sequence variants: a joint consensus recommendation of the American College of Medical Genetics and Genomics and the Association for Molecular Pathology. Genet Med. 2015;17(5):405-24. doi:10.1038/gim.2015.30.

33. Cingolani P, Patel VM, Coon M, Nguyen T, Land SJ, Ruden DM et al. Using Drosophila melanogaster as a Model for Genotoxic Chemical Mutational Studies with a New Program, SnpSift. Frontiers in genetics. 2012;3:35. doi:10.3389/fgene.2012.00035.

34. Stavnezer J, Guikema JE, Schrader CE. Mechanism and regulation of class switch recombination. Annual review of immunology. 2008;26:261-92. doi:10.1146/annurev.immunol.26.021607.090248.

35. Sirbu BM, Cortez D. DNA damage response: three levels of DNA repair regulation. Cold Spring Harbor perspectives in biology. 2013;5(8):a012724. doi:10.1101/cshperspect.a012724.

36. Nissenkorn A, Levy-Shraga Y, Banet-Levi Y, Lahad A, Sarouk I, Modan-Moses D. Endocrine abnormalities in ataxia telangiectasia: findings from a national cohort. Pediatric research. 2016;79(6):889.

37. Canny G, Roifman C, Weitzman S, Braudo M, Levison H. A pulmonary infiltrate in a child with ataxia telangiectasia. Annals of allergy. 1988;61(6).

38. Amirifar P, Yazdani R, Moeini Shad T, Ghanadan A, Abolhassani H, Lavin M et al. Cutaneous Granulomatosis and Class Switching Defect as a Presenting Sign in Ataxia-Telangiectasia: First Case from the National Iranian Registry and Review of the Literature. Immunol Invest. 2020;49(6):597-610. doi:10.1080/08820139.2019.1692864.

39. Staples ER, McDermott EM, Reiman A, Byrd PJ, Ritchie S, Taylor AM et al. Immunodeficiency in ataxia telangiectasia is correlated strongly with the presence of two null mutations in the ataxia telangiectasia mutated gene. Clinical and experimental immunology. 2008;153(2):214-20. doi:10.1111/j.1365-2249.2008.03684.x.

40. Etzioni A, Ben-Barak A, Peron S, Durandy A. Ataxia-telangiectasia in twins presenting as autosomal recessive hyper-immunoglobulin M syndrome. Isr Med Assoc J. 2007;9(5):406-7.

41. Aghamohammadi A, Imai K, Moazzami K, Abolhassani H, Tabatabaeiyan M, Parvaneh N et al. Ataxia-telangiectasia in a patient presenting with hyper-immunoglobulin M syndrome. J Investig Allergol Clin Immunol. 2010;20(5):442-5.

42. Rawat A, Imai K, Suri D, Gupta A, Bhisikar S, Saikia B et al. Ataxia Telangiectasia Masquerading as Hyper IgM Syndrome. Indian journal of pediatrics. 2016;83(3):270-1. doi:10.1007/s12098-015-1852-x.

43. Pan Q, Petit-Frere C, Lahdesmaki A, Gregorek H, Chrzanowska KH, Hammarstrom L. Alternative end joining during switch recombination in patients with ataxia-telangiectasia. European journal of immunology. 2002;32(5):1300-8. doi:10.1002/1521-4141(200205)32:5<1300::aid-immu1300>3.0.co;2-l.

44. Pan-Hammarstrom Q, Dai S, Zhao Y, van Dijk-Hard IF, Gatti RA, Borresen-Dale AL et al. ATM is not required in somatic hypermutation of VH, but is involved in the introduction of mutations in the switch mu region. J Immunol. 2003;170(7):3707-16.

45. Théry C, Amigorena S. The cell biology of antigen presentation in dendritic cells. Current opinion in immunology. 2001;13(1):45-51. doi:10.1016/s0952-7915(00)00180-1.

46. Kotsias F, Cebrian I, Alloatti A. Antigen processing and presentation. International review of cell and molecular biology. 2019;348:69-121. doi:10.1016/bs.ircmb.2019.07.005.

47. Joffre OP, Segura E, Savina A, Amigorena S. Cross-presentation by dendritic cells. Nature reviews Immunology. 2012;12(8):557-69. doi:10.1038/nri3254.

48. O'Leary JG, Goodarzi M, Drayton DL, von Andrian UH. T cell–and B cell–independent adaptive immunity mediated by natural killer cells. Nature immunology. 2006;7(5):507-16.

49. Basso K, Dalla-Favera R. Germinal centres and B cell lymphomagenesis. Nature Reviews Immunology. 2015;15(3):172-84.

50. Klein U, Dalla-Favera R. Germinal centres: role in B-cell physiology and malignancy. Nature Reviews Immunology. 2008;8(1):22-33.

51. Laman JD, Claassen E, Noelle RJ. Functions of CD40 and Its Ligand, gp39 (CD40L). Critical reviews in immunology. 2017;37(2-6):371-420. doi:10.1615/CritRevImmunol.v37.i2-6.100.

52. Chen L, Guo L, Tian J, Zheng B, Han S. Deficiency in activation-induced cytidine deaminase promotes systemic autoimmunity in lpr mice on a C57BL/6 background. Clinical and experimental immunology. 2010;159(2):169-75. doi:10.1111/j.1365-2249.2009.04058.x.

53. Reiman A, Srinivasan V, Barone G, Last JI, Wootton LL, Davies EG et al. Lymphoid tumours and breast cancer in ataxia telangiectasia; substantial protective effect of residual ATM kinase activity against childhood tumours. British journal of cancer. 2011;105(4):586-91. doi:10.1038/bjc.2011.266.

54. Suarez F, Mahlaoui N, Canioni D, Andriamanga C, Dubois d'Enghien C, Brousse N et al. Incidence, presentation, and prognosis of malignancies in ataxia-telangiectasia: a report from the French national registry of primary immune deficiencies. Journal of clinical oncology : official journal of the American Society of Clinical Oncology. 2015;33(2):202-8. doi:10.1200/jco.2014.56.5101.

55. Morrell D, Cromartie E, Swift M. Mortality and cancer incidence in 263 patients with ataxia-telangiectasia. Journal of the National Cancer Institute. 1986;77(1):89-92.

56. Taylor A, Metcalfe J, Thick J, Mak Y. Leukemia and lymphoma in ataxia telangiectasia. Blood. 1996;87(2):423-38.

57. Rothblum-Oviatt C, Wright J, Lefton-Greif MA, McGrath-Morrow SA, Crawford TO, Lederman HM. Ataxia telangiectasia: a review. Orphanet journal of rare diseases. 2016;11(1):159.

58. Alvarez-Quilon A, Serrano-Benitez A, Lieberman JA, Quintero C, Sanchez-Gutierrez D, Escudero LM et al. ATM specifically mediates repair of double-strand breaks with blocked DNA ends. Nature communications. 2014;5:3347. doi:10.1038/ncomms4347.

59. Bednarski JJ, Sleckman BP. Lymphocyte development: integration of DNA damage response signaling. Advances in immunology. 2012;116:175-204. doi:10.1016/b978-0-12-394300-2.00006-5.

60. Cannan WJ, Pederson DS. Mechanisms and Consequences of Double-Strand DNA Break Formation in Chromatin. Journal of cellular physiology. 2016;231(1):3-14. doi:10.1002/jcp.25048.

61. Hasham MG, Snow KJ, Donghia NM, Branca JA, Lessard MD, Stavnezer J et al. Activation-induced cytidine deaminase-initiated off-target DNA breaks are detected and resolved during S phase. J Immunol. 2012;189(5):2374-82. doi:10.4049/jimmunol.1200414.

62. Yamane A, Robbiani DF, Resch W, Bothmer A, Nakahashi H, Oliveira T et al. RPA accumulation during class switch recombination represents 5'-3' DNA-end resection during the S-G2/M phase of the cell cycle. Cell Rep. 2013;3(1):138-47. doi:10.1016/j.celrep.2012.12.006.

63. Di Virgilio M, Callen E, Yamane A, Zhang W, Jankovic M, Gitlin AD et al. Rif1 prevents resection of DNA breaks and promotes immunoglobulin class switching. Science. 2013;339(6120):711-5. doi:10.1126/science.1230624.

64. Saha T, Sundaravinayagam D, Di Virgilio M. Charting a DNA Repair Roadmap for Immunoglobulin Class Switch Recombination. Trends Biochem Sci. 2021;46(3):184-99. doi:10.1016/j.tibs.2020.10.005.

65. Lee K, Ji JH, Yoon K, Che J, Seol JH, Lee SE et al. Microhomology Selection for Microhomology Mediated End Joining in Saccharomyces cerevisiae. Genes (Basel). 2019;10(4). doi:10.3390/genes10040284.

66. Soni A, Siemann M, Grabos M, Murmann T, Pantelias GE, Iliakis G. Requirement for Parp-1 and DNA ligases 1 or 3 but not of Xrcc1 in chromosomal translocation formation by backup end joining. Nucleic acids research. 2014;42(10):6380-92. doi:10.1093/nar/gku298.

67. Caddle LB, Hasham MG, Schott WH, Shirley BJ, Mills KD. Homologous recombination is necessary for normal lymphocyte development. Mol Cell Biol. 2008;28(7):2295-303. doi:10.1128/mcb.02139-07.

68. Masson JY, Tarsounas MC, Stasiak AZ, Stasiak A, Shah R, McIlwraith MJ et al. Identification and purification of two distinct complexes containing the five RAD51 paralogs. Genes Dev. 2001;15(24):3296-307. doi:10.1101/gad.947001.

69. Hasham MG, Donghia NM, Coffey E, Maynard J, Snow KJ, Ames J et al. Widespread genomic breaks generated by activation-induced cytidine deaminase are prevented by homologous recombination. Nat Immunol. 2010;11(9):820-6. doi:10.1038/ni.1909.

70. Winsey SL, Haldar NA, Marsh HP, Bunce M, Marshall SE, Harris AL et al. A variant within the DNA repair gene XRCC3 is associated with the development of melanoma skin cancer. Cancer research. 2000;60(20):5612-6.

71. Das R, Kundu S, Laskar S, Choudhury Y, Ghosh SK. Assessment of DNA repair susceptibility genes identified by whole exome sequencing in head and neck cancer. DNA repair. 2018;66-67:50-63. doi:10.1016/j.dnarep.2018.04.005.

72. Lavin MF, Delia D, Chessa L. ATM and the DNA damage response: Workshop on Ataxia‐Telangiectasia and Related Syndromes. EMBO reports. 2006;7(2):154-60.

**Figure legends**

**Figure 1-** Flow chart of data analysis and filtering steps for identification of genetic variants in the 20 A-T patients by WES analysis.

**Figure 2-** The results of GO, KEGG and Jensen enrichment analysis on 1074 candidate genes. A) The top 10 enriched GO cellular components for candidate genes. B) The top 10 enriched KEGG pathways for candidate genes. C) The top 10 enriched Jensen diseases for candidate genes.

**Figure 3-** The position (A) and frequency in A-T patients (B) and frequency in the normal population (C) of XRCC3 rs861539 polymorphism across A-T patients with CSR-N and CSR-D phenotypes. CADD: Combined Annotation Dependent Depletion a tool for scoring the deleteriousness of single nucleotide variants. MSC: mutation significance cutoff the lowest expected monogenic disorder based on CADD cutoff value for the specific gene.

**
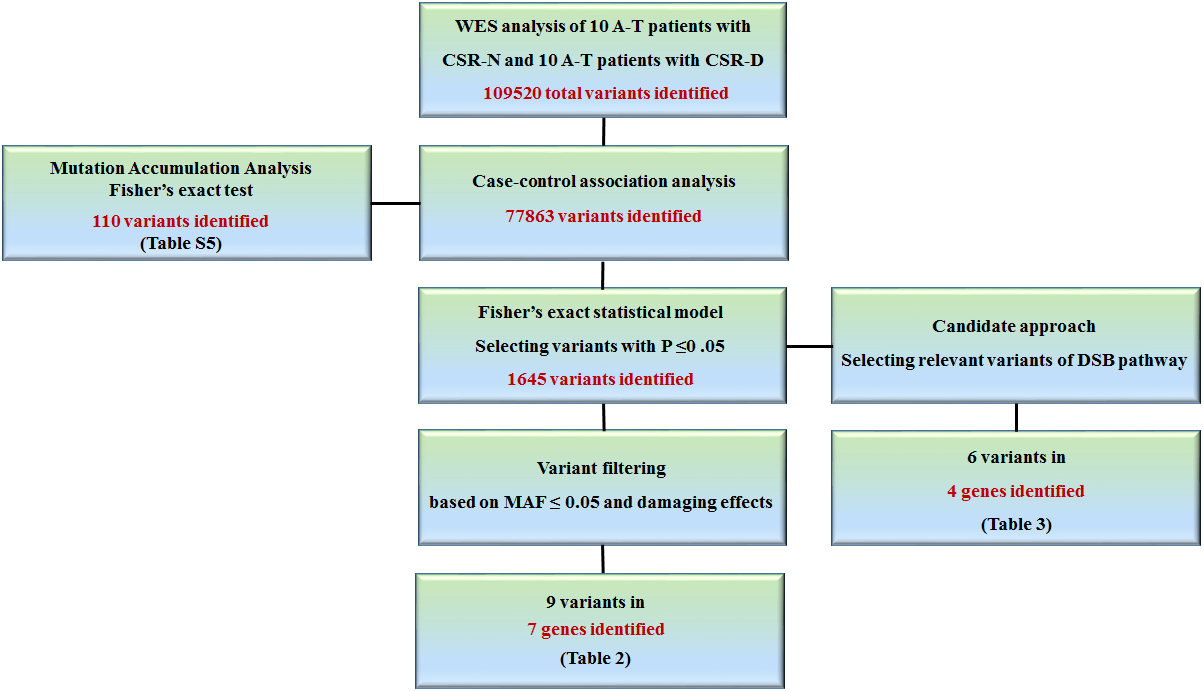
Figure 1-**


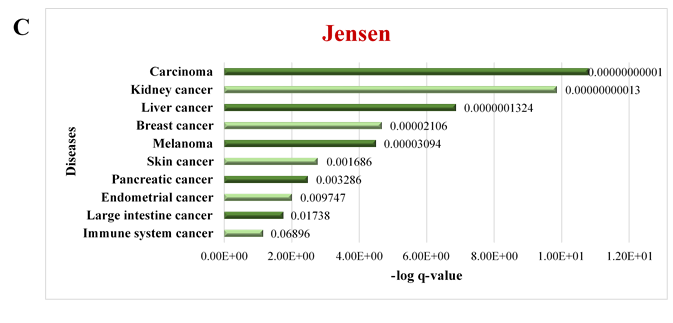

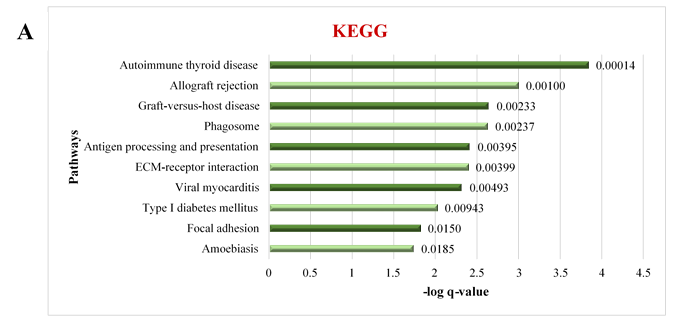

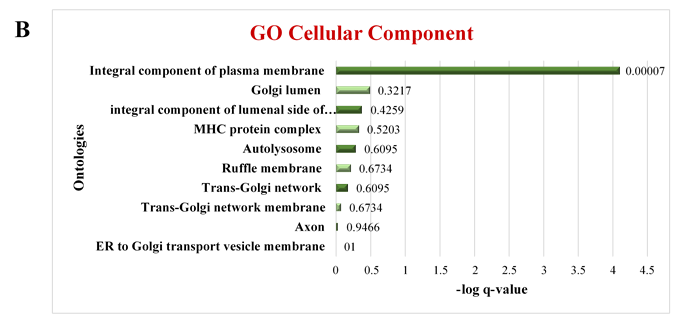
**Figure 2-**

**C**

**B**

**A**

**A**

**C**

**B**

**Figure 3-**

**
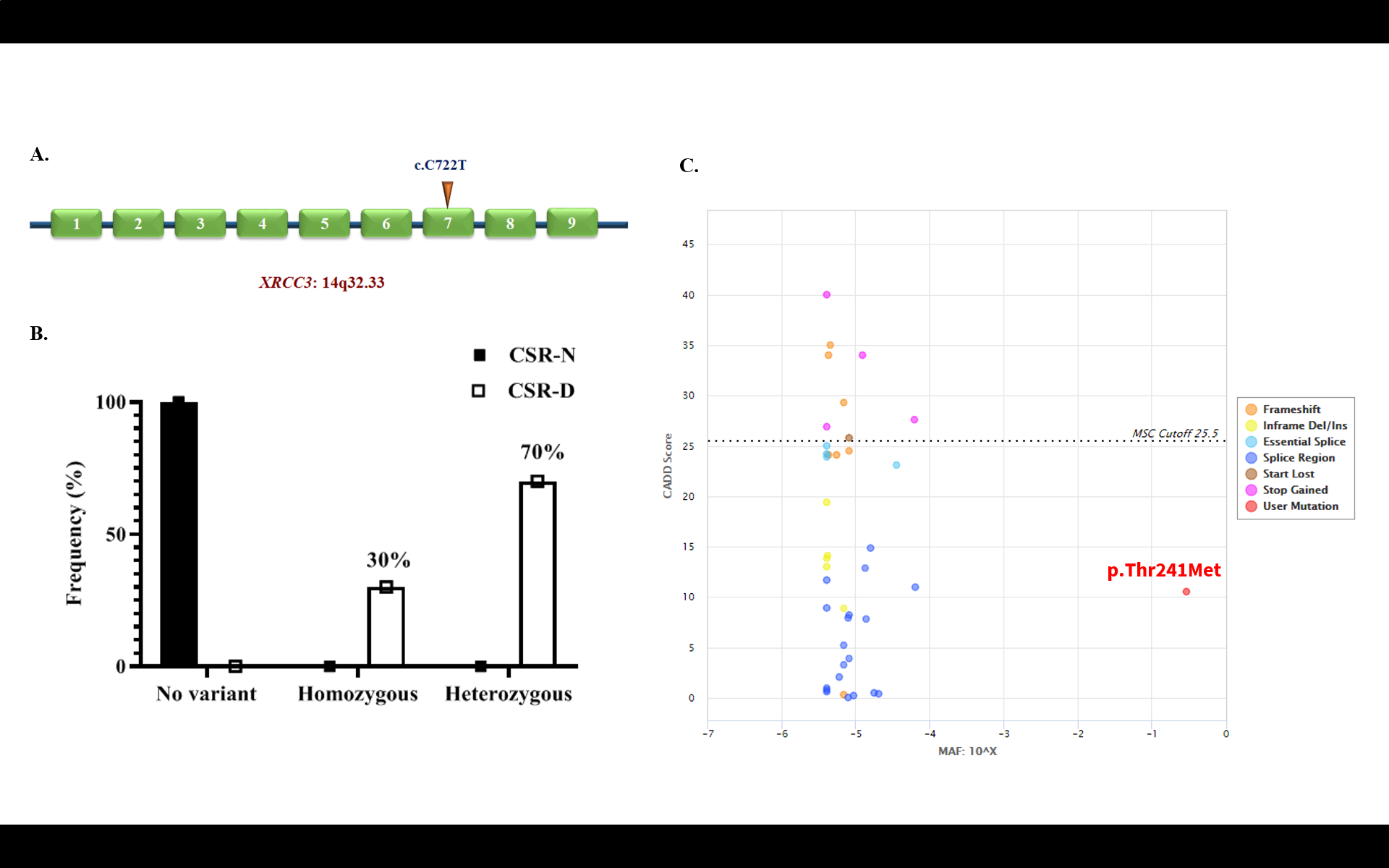
**

| **Parameter** | **Total patients (n=20)** | **Patients with CSR-N (n=10)** | **Patients with CSR-D (n=10)** | ***P-value*** |
| --- | --- | --- | --- | --- |
| Age at the study time, years (IQR) | 9.0 (7.25-10.7) | 8.5 (6.25-11.7) | 9.0 (7.25-11.0) | 0.241 |
| Age at diagnosis, years (IQR) | 5.0 (4.25-7.7) | 5.5 (4.0-7.2) | 4.8 (4.0-7.0) | 0.432 |
| Age at onset of ataxia, years (IQR) | 1.2 (0.75-2.0) | 1.0 (0.8-2.3) | 1.0 (0.8-2.3) | 0.324 |
| Age at onset of Infection, years (IQR) | 1.8 (1.0-2.8) | 2.0 (1.0-2.25) | 1.5 (1.25-1.6) | 0.371 |
| Age at onset of telangiectasia, years (IQR) | 4.0 (2.0-6.0) | 4.0 (2.0-6.8) | 3.8 (1.75-6.0) | 0.223 |
| Delay diagnosis, years (IQR) | 3.5 (1.0-5.2) | 3.7 (1.25-5.5) | 3.0 (1.0-5.0) | 0.724 |
| Sex, N (%)  Male  Female | 9 (45.0)  11 (55.0) | 7 (70.0)  3 (30.0) | 2 (20.0)  8 (80.0) | 0.035* |
| Consanguinity, N (%) | 16 (80.0) | 8 (80.0) | 8 (80.0) | 0.752 |
| Mortality, N (%)  Alive  Dead | 17 (85.0)  3 (15.0) | 9 (90.0)  1 (10.0) | 8 (80.0)  2 (20.0) | 0.221 |
| Infections (%) | 12 (60) | 3 (30) | 9 (90) | 0.020* |
| Respiratory infection (%) | 10 (50) | 2 (20) | 8 (80) | 0.025* |
| Diarrhea (%) | 7 (35) | 1 (10) | 6 (60) | 0.057 |
| Skin manifestation (%) | 6 (30) | 2 (20) | 4 (40) | 0.628 |
| Hepatosplenomegaly (%) | 7 (35) | 1 (10) | 6 (60) | 0.057 |
| Autoimmunity (%) | 3 (15) | 1 (10) | 2 (20) | 0.531 |
| Malignancy (%) | 1 (5) | 0 (0) | 1 (10) | 0.305 |
| AFP, ng/ml (IQR) | 125.0 (95.0-300.0) | 121.0 (91.0-301.2) | 126.0 (95.0-303.0) | 0.142 |
| IgG, mg/dl (IQR) | 740 (460.0-1180.0) | 840.0 (522.2-990.7) | 90.5 (21.0-215.7) | <0.001* |
| IgG1 (mg/dl) | 652.0 (98.0-773.0) | 767.0 (737.2-1019.5) | 98.0 (65.0-347.0) | <0.001* |
| IgG2 (mg/dl) | 36.0 (28.0-97.0) | 65.5 (29.75-153.25) | 32.0 (20.0-82.0) | 0.189 |
| IgG3 (mg/dl) | 33.0 (7.0-74.0) | 68.5 (31.0.-83.5) | 7 .0 (4.0-33.0) | 0.009* |
| IgG4 (mg/dl) | 4.0 (1.0-19.0) | 15.0 (4.5-28.5) | 1.0 (1.0-4.0) | 0.004* |
| IgA, mg/dl (IQR) | 25.0 (0-95.5) | 58.5 (31.0-100.7) | 4.5 (2.25-7.0) | 0.001* |
| IgM, mg/dl (IQR) | 175.0 (118.0-420.0) | 97.0 (61.0-168.7) | 428.0 (135.2-630.2) | 0.002* |
| IgE, IU/ml (IQR) | 3.0 (1.0-8.0) | 3.0 (1.75-9.0) | 2.8 (1.0-7.75) | 0.153 |
| *Ig, immunoglobulin; IQR, interquartile range rang, AFP: Alpha fetoprotein.*  *Normal ranges for AFP: < 20 ng/ml*  *Normal ranges for IgG: 500-1300 (mg/dl)*  *Normal ranges for IgG1: 280-1120 (mg/dl)*  *Normal ranges for IgG2: 30-630 (mg/dl)*  *Normal ranges for IgG3: 40-250(mg/dl)*  *Normal ranges for IgG4: 11-620 (mg/dl)*  *Normal ranges for IgA: <1 m: 7-94; 1 m to 12 m: 10-131; 1 y to 3 y:19-220; 4 y to 5 y: 48-345; 6 y to 7 y: 41-297; 8 y to 10 y: 51-297; 11 y to 13 y: 44-395, Adults: 70-400 (mg/dl).*  *Normal ranges for IgM: 1 m to 3 m12-87; 4m to 6 m: 25-120; 7 m to 12 m: 36-104; 1 y to 11 y: 55-210; Adults: 40-230 (mg/dl).*  *Normal ranges for IgE: <144 (IU/ml).*  ** P-value < 0.05 is statistically significant.* | | | | |

**Table 1-** Demographic, clinical and laboratory features between A-T patients with CSR-N and A-T patients with CSR-D.

**Table 2-** Identification of significant variants based on MAF and SIFT/CADD criteria between A-T patients with CSR-N and A-T patients with CSR-D. Hom: homozygous, Het: heterozygous.

| **Gene** | **Chr** | **Pos** | **dbSNP ID** | **Ref** | **Alt** | **Exonic Func.** | **Nuc/AA Change** | **MAF** | **Cases**  **(CSR-D)** | **Controls**  **(CSR-N)** |
| --- | --- | --- | --- | --- | --- | --- | --- | --- | --- | --- |
| ***Protective*** | | | | | | | | | | |
| *HLA-DRB5* | 6 | 32522172 | rs1136744 | G | A | missense | c.C103T/ p.R35C | 0.0436 | - | 3 Hom |
| *KIR3DL1* | 19 | 54819832 | rs139070113 | G | T | missense | c.G475T/ p.G159W | 0 | - | 2 Hom, 2 Het |
|  |  | 54818479 | rs62124092 | A | G | missense | c.A235G/ p.S79G | 0 | - | 2 Hom, 3 Het |
| *GOLGA8J* | 15 | 30093429 | rs201797381 | A | C | missense | c.A1829C/ p.H610P | 0.018 | - | 2 Hom, 1 Het |
| *MUC6* | 11 | 1016779 | . | G | A | missense | c.C6022T/ p.H2008Y | 0 | 1 Het | 4 Het |
| ***Risk*** | | | | | | | | | | |
| *GXYLT1* | 12 | 42087868 | rs200973030 | C | T | missense | c.G1148A/ p.C383Y | 0.0003 | 7 Het | 1 Het |
|  |  | 42087869 | rs202200134 | A | G | missense | c.T1147C/ p.C383R | 0.0003 | 7 Het | 1 Het |
| *MUC4* | 3 | 195779671 | rs200412534 | G | T | missense | c.C11909A/ p.P3970H | 0.0013 | 5 Het | 2 Het |
| *VWA3B* | 2 | 98311966 | rs17428626 | C | G | missense | c.C2640G/ p.D880E | 0.0258 | 1 Hom, 2 Het | 1 Het |

**Table 3-** Identification of significant variants in DSB response pathway between A-T patients with CSR-N and A-T patients with CSR-D. Hom: homozygous, Het: heterozygous.

| **Gene** | **Chr** | **Pos** | **dbSNP ID** | **Ref** | **Alt** | **Exonic Func.** | **Nuc/AA Change** | **MAF** | **Cases**  **(CSR-D)** | **Controls**  **(CSR-N)** |
| --- | --- | --- | --- | --- | --- | --- | --- | --- | --- | --- |
| ***Protective*** | | | | | | | | | | |
| *FANCM* | 14 | 45175386 | rs1367580 | G | T | missense | c.G2554T/ p.V852L | 0.1835 | 2 Het | 1 Hom, 5 Het |
|  |  | 45181697 | rs78211950 | A | G | missense | c.A4300G/ p.I1434V | 0.1849 | 2 Het | 1 Hom, 3 Het |
|  |  | 45196265 | rs3736772 | C | G | missense | c.C5356G/ p.P1786A | 0.2009 | 2 Het | 1 Hom, 3 Het |
| ***Risk*** | | | | | | | | | | |
| *RAD23B* | 9 | 107322047 | rs1805329 | C | T | missense | c.C683T/ p.A228V | 0.1858 | 1 Hom, 3 Het | - |
| *MLH1* | 3 | 37012077 | rs1799977 | A | G | missense | c.A655G/ p.I219V | 0.1355 | 6 Het | - |
| *XRCC3* | 14 | 103699416 | rs861539 | G | A | missense | c.C722T/ p.T241M | 0.2435 | 3 Hom, 7 Het | - |

**Supplementary Data**

**Genetic Risk Variants for Class Switching Recombination Defects in Ataxia-Telangiectasia Patients**

Amirifar et al.


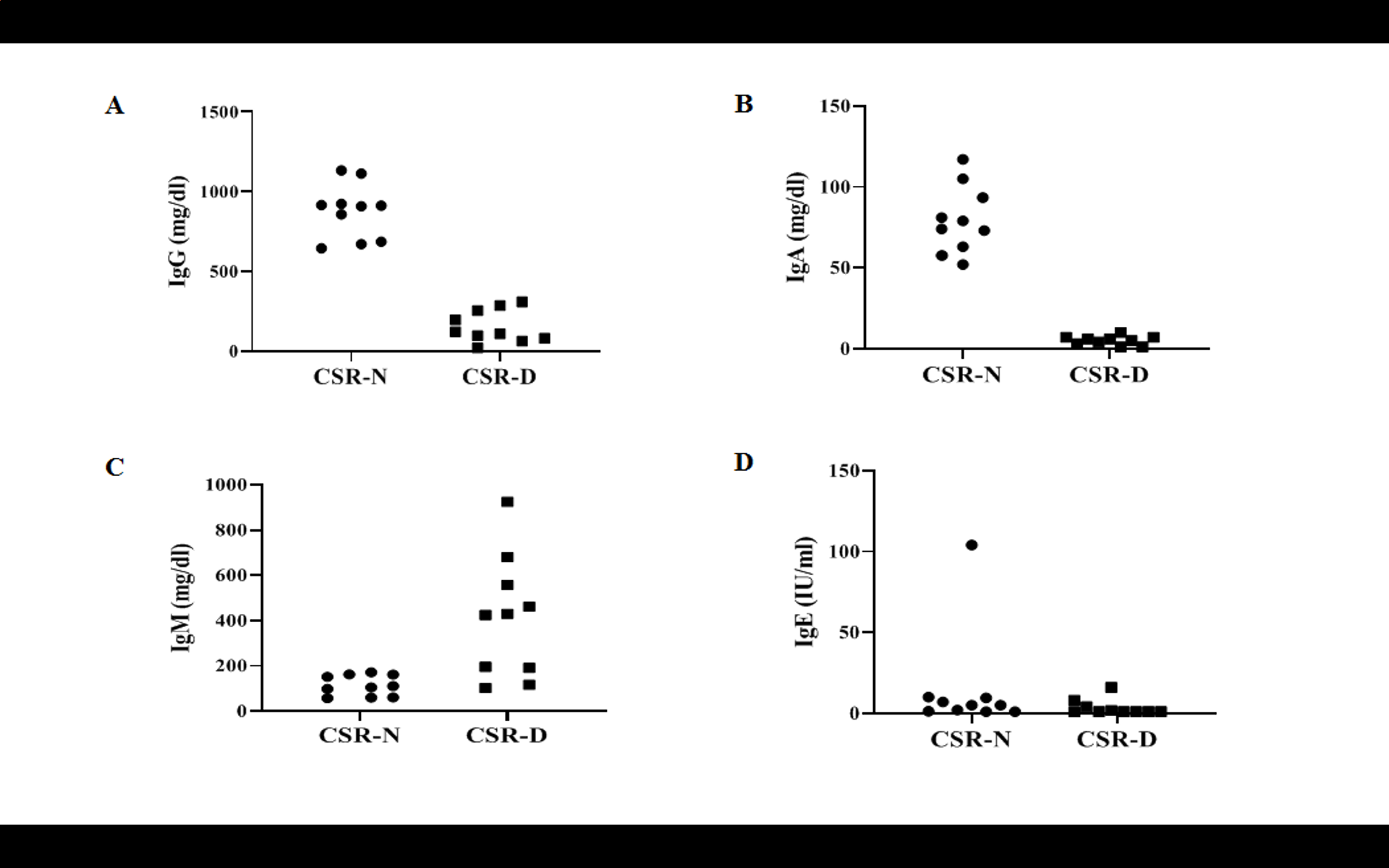


**Figure S1-** The distribution of immunoglobulin levels for each patient in CSR-N and CSR-D.

**Figure S2-** *In vitro* IgE production level of the study group. Cell culture was used to evaluate the capabilities of CSR toward IgE production. IgE production from cultured cells was found to be reduced in patients with CSR defect profiles when compared to controls (*p*<0.001).

**Figure S3-** Examples from nested amplification of the recombinant Sµ/Sα junctions of one A-T patient with normal Ig level (A) and one A-T patient with CSR defect (B). The number of Sμ-Sα fragments was determined from 10 PCR reactions to run in parallel (lanes 1-10), using genomic DNA samples from the same individual. The amplification of bands with different sizes is due to the polyclonal rearrangement at the Sµ/Sα region. M, Marker; NC, Negative control (No DNA); 1-10, A-T patient.


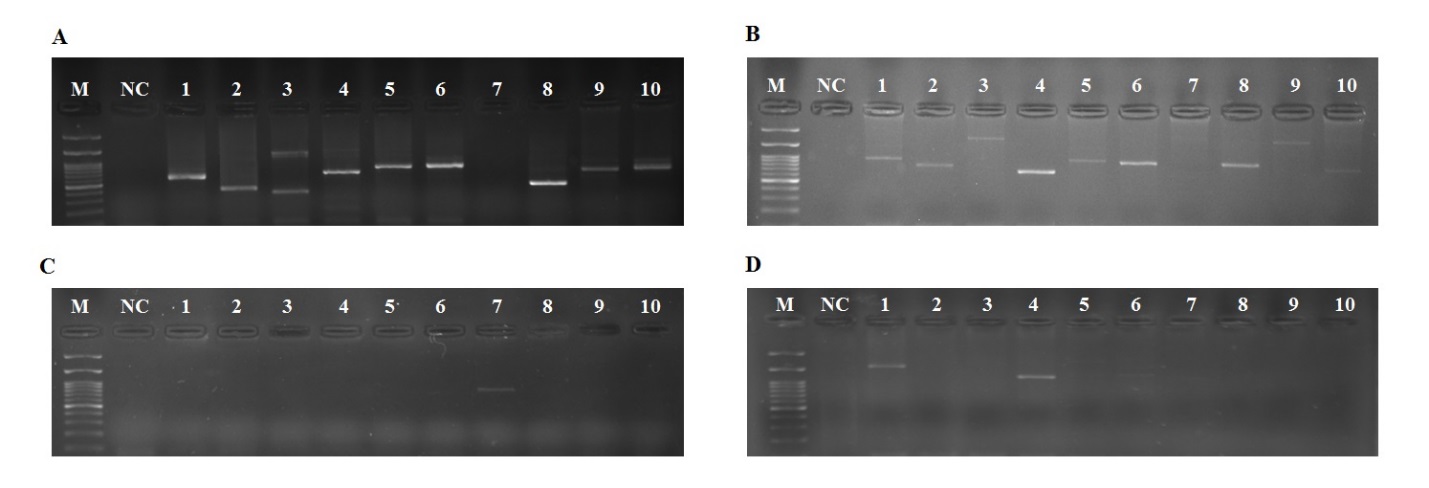


**A**

**B**

**
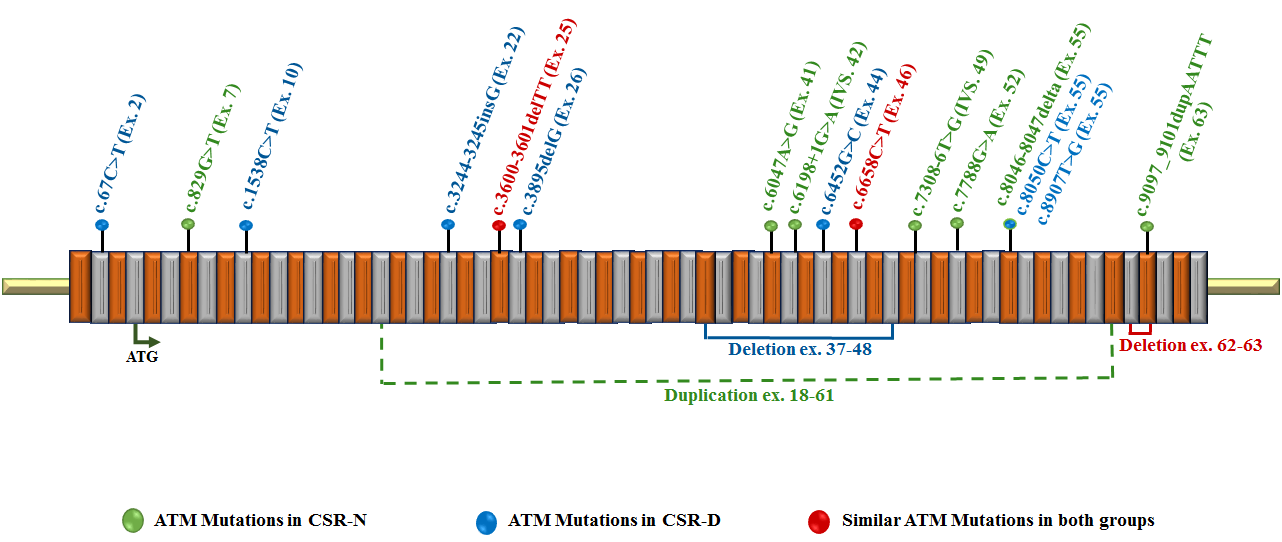
Figure S4-** Diversity of *ATM* gene mutations detected in CSR-N and CSR-D patients.

**Table S1.** *ATM* mutations of patients with ataxia-telangiectasia.

| **Variant classification** | **Chr. position (hg19)** | **Variant location** | **A.A change** | **ATM mutation** | **Zygosity** | **Code** |
| --- | --- | --- | --- | --- | --- | --- |
| Pathogenic | 108235800-108236250 | Exon 62-63 | - | Deletion exon 62-63 | Homozygous | CSR-D1 |
| Pathogenic | 108098418 | Exon 2 | p.R23* | c.67C>T | Homozygous | CSR-D2 |
| Pathogenic | 108121729  108205735 | Exon 10  Exon 55 | p.Q513*  p.Q2689* | c.1537C>T  c.8050C>T | Compound heterozygous | CSR-D3 |
| Pathogenic | 108143539 | Exon 22 | p.H1082Rfs*14 | c.3244-3245insG | Homozygous | CSR-D4 |
| Pathogenic | 108155101 | Exon 26 | p.A1299Pfs*50 | c.3895delG | Homozygous | CSR-D5 |
| Likely pathogenic | 108196122 | Exon 46 | p.Q2220* | c.6658C>T | Homozygous | CSR-D6 |
| Pathogenic | 108153459 | Exon 25 | p.F1201Wfs*3 | c.3600-3601delTT | Homozygous | CSR-D7 |
| Pathogenic | 108205735  108235865 | Exon 55  Exon 62 | p.Q2684*  p.Q2969* | c.8907T>G  c.8050C>T | Compound heterozygous | CSR-D8 |
| Likely pathogenic | 108190785 | Exon 44 | p.R2151T | c.6452G>C | Homozygous | CSR-D9 |
| Pathogenic | 108175400-108198490 | Exon 37-48 | - | Deletion exon 37-48 | Homozygous | CSR-D10 |
| Pathogenic  Likely pathogenic | 108186841  108186590 | Intron 42  Exon 41 | Splice donor  p.D2016G | c.6198+1G>A  c.6047A>G | Compound heterozygous | CSR-N1 |
| Pathogenic  Likely pathogenic | -  108202764 | Exon 18-61  Exon 52 | -  p.E2596= | Duplication exon 18-61  c.7788G>A | Compound heterozygous | CSR-N2 |
| Pathogenic | 108236160 | Exon 63 | p.L3035Ifs*8 | c.9097_9101dupAATTT | Homozygous | CSR-N3 |
| Pathogenic | 108200935 | Intron 49 | - | c.7308-6T>G | Homozygous | CSR-N4 |
| Pathogenic | 108205730 | Exon 55 | p.I2683Tfs*4 | c.8046-8047delTA | Homozygous | CSR-N5 |
| Pathogenic | 108196122 | Exon 46 | p.Q2220* | c.6658C>T | Homozygous | CSR-N6 |
| Pathogenic | 108115681 | Exon 7 | p.Glu277* | c.829G>T | Homozygous | CSR-N7 |
| Pathogenic | 108196122 | Exon 46 | p.Q2220* | c.6658C>T | Homozygous | CSR-N8 |
| Pathogenic | 108235800-108236250 | Exon 62-63 | - | Deletion exon 62-63 | Homozygous | CSR-N9 |
| Pathogenic | 108153460-108153461 | Exon 25 | p.F1201Wfs*3 | c.3600_3601delTT | Homozygous | CSR-N10 |

*AA: amino acid, Chr: chromosome, EX: exon.*

**Table S2-** The top 10 enriched cellular component items for candidate genes.

| **Index** | **Name** | ***P*-value** | **Adjusted *p*-value** | **Odds Ratio** | **Combined score** |
| --- | --- | --- | --- | --- | --- |
| 1 | Integral component of plasma membrane (GO:0005887) | 1.763e-7 | 0.00007864 | 1.58 | 24.61 |
| 2 | Golgi lumen (GO:0005796) | 0.002164 | 0.3217 | 2.48 | 15.20 |
| 3 | Integral component of lumenal side of endoplasmic reticulum membrane (GO:0071556) | 0.003820 | 0.4259 | 3.86 | 21.51 |
| 4 | MHC protein complex (GO:0042611) | 0.002081 | 0.4641 | 5.19 | 32.03 |
| 5 | Autolysosome (GO:0044754) | 0.006999 | 0.5203 | 7.00 | 34.75 |
| 6 | Ruffle membrane (GO:0032587) | 0.006833 | 0.6095 | 2.82 | 14.05 |
| 7 | Trans-Golgi network (GO:0005802) | 0.01057 | 0.6734 | 1.82 | 8.27 |
| 8 | Trans-Golgi network membrane (GO:0032588) | 0.01525 | 0.8500 | 2.20 | 9.19 |
| 9 | Axon (GO:0030424) | 0.01910 | 0.9466 | 1.85 | 7.34 |
| 10 | ER to Golgi transport vesicle membrane (GO:0012507) | 0.02440 | 1.000 | 2.42 | 8.99 |

**Table S3-** The top 10 enriched KEGG pathway items for candidate genes.

| **Index** | **Name** | ***P*-value** | **Adjusted *p*-value** | **Odds Ratio** | **Combined score** |
| --- | --- | --- | --- | --- | --- |
| 1 | Autoimmune thyroid disease | 0.00001698 | 0.005229 | 4.26 | 46.75 |
| 2 | Allograft rejection | 0.0001327 | 0.02044 | 4.45 | 39.74 |
| 3 | Graft-versus-host disease | 0.0002471 | 0.02537 | 4.13 | 34.27 |
| 4 | Phagosome | 0.0004633 | 0.03567 | 2.35 | 18.04 |
| 5 | Antigen processing and presentation | 0.0007223 | 0.04449 | 2.93 | 21.19 |
| 6 | Viral myocarditis | 0.001013 | 0.05198 | 3.19 | 21.97 |
| 7 | ECM-receptor interaction | 0.001277 | 0.05618 | 2.75 | 18.33 |
| 8 | Type I diabetes mellitus | 0.001717 | 0.06612 | 3.50 | 22.27 |
| 9 | Complement and coagulation cascades | 0.002997 | 0.1025 | 2.62 | 15.21 |
| 10 | Salivary secretion | 0.008140 | 0.2507 | 2.30 | 11.05 |

**Table S4-** The top 10 enriched Jensen disease items for candidate genes.

| **Index** | **Name** | ***p*-value** | **Adjusted *p*-value** | **Odds Ratio** | **Combined score** |
| --- | --- | --- | --- | --- | --- |
| 1 | Carcinoma | 8.466e-15 | 1.533e-11 | 1.20 | 38.81 |
| 2 | Kidney cancer | 1.496e-13 | 1.355e-10 | 1.60 | 47.38 |
| 3 | Liver cancer | 2.193e-10 | 1.324e-7 | 2.22 | 49.39 |
| 4 | Breast cancer | 4.652e-8 | 0.00002106 | 2.24 | 37.78 |
| 5 | Melanoma | 8.542e-8 | 0.00003094 | 2.11 | 34.31 |
| 6 | Skin cancer | 0.000005584 | 0.001686 | 1.97 | 23.88 |
| 7 | Pancreatic cancer | 0.00001270 | 0.003286 | 2.86 | 32.27 |
| 8 | Endometrial cancer | 0.00004306 | 0.009747 | 2.11 | 21.22 |
| 9 | Large intestine cancer | 0.00008640 | 0.01738 | 2.76 | 25.78 |
| 10 | Immune system cancer | 0.0003808 | 0.06896 | 2.45 | 19.32 |

**Table S5-** The top 10 enriched biological process items for overlapping genes between CSR-N and CSR-D.

| **Index** | **Name** | ***p*-value** | **Adjusted *p*-value** | **Odds Ratio** | **Combined score** |
| --- | --- | --- | --- | --- | --- |
| 1 | External encapsulating structure organization (GO:0045229) | 0.0000002528 | 0.001070 | 2.69 | 40.88 |
| 2 | Extracellular structure organization (GO:0043062) | 0.000001612 | 0.003411 | 2.54 | 33.91 |
| 3 | Extracellular matrix organization (GO:0030198) | 0.00004072 | 0.05744 | 2.04 | 20.57 |
| 4 | Substrate adhesion-dependent cell spreading (GO:0034446) | 0.00007895 | 0.08353 | 4.25 | 40.14 |
| 5 | Regulation of actin cytoskeleton reorganization (GO:2000249) | 0.0001110 | 0.09394 | 4.84 | 44.04 |
| 6 | Synapse organization (GO:0050808) | 0.0004620 | 0.2444 | 2.42 | 18.62 |
| 7 | Neuromuscular junction development (GO:0007528) | 0.0004066 | 0.2444 | 5.71 | 44.56 |
| 8 | Negative regulation of toll-like receptor signaling pathway (GO:0034122) | 0.0004182 | 0.2444 | 4.39 | 34.18 |
| 9 | Negative regulation of developmental growth (GO:0048640) | 0.0007319 | 0.3442 | 4.47 | 32.26 |
| 10 | Regulation of toll-like receptor 9 signaling pathway (GO:0034163) | 0.001048 | 0.3609 | 9.50 | 65.18 |

**Table S6-** The top 10 enriched molecular function items for overlapping genes between CSR-N and CSR-D.

| **Index** | **Name** | ***p*-value** | **Adjusted *p*-value** | **Odds Ratio** | **Combined score** |
| --- | --- | --- | --- | --- | --- |
| 1 | Glycerophospholipid flippase activity (GO:0140333) | 0.00001227 | 0.01068 | 15.98 | 180.71 |
| 2 | Alpha-1,4-glucosidase activity (GO:0004558) | 0.0001992 | 0.04204 | 45.58 | 388.44 |
| 3 | Phosphatidylcholine flippase activity (GO:0140345) | 0.0001992 | 0.04204 | 45.58 | 388.44 |
| 4 | Phosphatidylinositol trisphosphate phosphatase activity (GO:0034594) | 0.0001992 | 0.04204 | 45.58 | 388.44 |
| 5 | Transmembrane receptor protein tyrosine kinase activity (GO:0004714) | 0.0002416 | 0.04204 | 3.48 | 28.99 |
| 6 | Alpha-glucosidase activity (GO:0090599) | 0.0005591 | 0.07610 | 22.79 | 170.69 |
| 7 | Voltage-gated chloride channel activity (GO:0005247) | 0.0006123 | 0.07610 | 11.40 | 84.35 |
| 8 | Vascular endothelial growth factor-activated receptor activity (GO:0005021) | 0.001221 | 0.1328 | 15.19 | 101.92 |
| 9 | Low-density lipoprotein particle binding (GO:0030169) | 0.001570 | 0.1366 | 6.22 | 40.16 |
| 10 | Olfactory receptor activity (GO:0004984) | 0.001500 | 0.1366 | 1.92 | 12.50 |

**Table S7-** The top 10 enriched cellular component items for overlapping genes between CSR-N and CSR-D.

| **Index** | **Name** | ***p*-value** | **Adjusted *p*-value** | **Odds Ratio** | **Combined score** |
| --- | --- | --- | --- | --- | --- |
| 1 | Collagen-containing extracellular matrix (GO:0062023) | 0.000004089 | 0.001497 | 2.04 | 25.35 |
| 2 | Integral component of plasma membrane (GO:0005887) | 0.00009078 | 0.01661 | 1.42 | 13.18 |
| 3 | Cytoplasmic vesicle membrane (GO:0030659) | 0.0002377 | 0.02900 | 1.79 | 14.91 |
| 4 | Endoplasmic reticulum lumen (GO:0005788) | 0.0004363 | 0.03992 | 1.88 | 14.53 |
| 5 | Sarcolemma (GO:0042383) | 0.0007347 | 0.05378 | 3.43 | 24.74 |
| 6 | Lysosomal lumen (GO:0043202) | 0.001321 | 0.08056 | 2.61 | 17.33 |
| 7 | Vesicle membrane (GO:0012506) | 0.001916 | 0.1002 | 2.71 | 16.97 |
| 8 | Basement membrane (GO:0005604) | 0.002550 | 0.1025 | 3.06 | 18.30 |
| 9 | Intermediate filament cytoskeleton (GO:0045111) | 0.002801 | 0.1025 | 2.49 | 14.61 |
| 10 | Asymmetric synapse (GO:0032279) | 0.002289 | 0.1025 | 2.15 | 13.05 |

**Table S8-** The top 10 enriched KEGG pathway items for overlapping genes between CSR-N and CSR-D.

| **Index** | **Name** | ***p*-value** | **Adjusted *p*-value** | **Odds Ratio** | **Combined score** |
| --- | --- | --- | --- | --- | --- |
| 1 | ECM-receptor interaction | 0.000001338 | 0.0004069 | 3.83 | 51.77 |
| 2 | Protein digestion and absorption | 0.0001942 | 0.02952 | 2.76 | 23.60 |
| 3 | Galactose metabolism | 0.002622 | 0.2046 | 3.97 | 23.59 |
| 4 | ABC transporters | 0.002693 | 0.2046 | 3.26 | 19.30 |
| 5 | Olfactory transduction | 0.003683 | 0.2239 | 1.54 | 8.64 |
| 6 | Lysosome | 0.007125 | 0.3610 | 1.99 | 9.86 |
| 7 | Antigen processing and presentation | 0.009369 | 0.3792 | 2.28 | 10.67 |
| 8 | Dilated cardiomyopathy | 0.009980 | 0.3792 | 2.12 | 9.75 |
| 9 | Fatty acid elongation | 0.01871 | 0.5688 | 3.26 | 12.96 |
| 10 | Pancreatic secretion | 0.01695 | 0.5688 | 1.97 | 8.03 |

**Table S9-** Gene list of mutation accumulation analysis

| **Gene** | **Fisher *p*-value** | ***q*-value** | **Non.ref Allele.diff** |
| --- | --- | --- | --- |
| ***MUC5B*** | **1.07E-15** | **8.52E-12** | **227** |
| ***FLG*** | **8.08E-16** | **8.52E-12** | **-161** |
| *SIRPB1* | 6.90E-14 | 3.67E-10 | -128 |
| ***MUC4*** | **8.78E-11** | **3.50E-07** | **-400** |
| *SERPINB10* | 4.44E-10 | 1.36E-06 | 32 |
| *TAS2R43* | 5.11E-10 | 1.36E-06 | -159 |
| ***CD276*** | **8.15E-10** | **1.86E-06** | **29** |
| ***MUC19*** | **6.57E-09** | **1.31E-05** | **-68** |
| *NT5C3B* | 7.86E-09 | 1.39E-05 | -29 |
| *RLIM* | 1.32E-08 | 2.11E-05 | -33 |
| ***SIRPA*** | **3.68E-08** | **5.33E-05** | **-73** |
| ***MOV10*** | **4.43E-08** | **5.88E-05** | **30** |
| *KRTAP10-7* | 7.23E-08 | 8.86E-05 | 41 |
| *ZNF417* | 1.89E-07 | 0.000215 | 36 |
| *OR51M1* | 3.86E-07 | 0.00038 | 44 |
| *KRTAP5-5* | 4.28E-07 | 0.00038 | -81 |
| *PCDHB16* | 3.95E-07 | 0.00038 | 74 |
| *OR2T4* | 4.29E-07 | 0.00038 | -56 |
| ***HLA-B*** | **5.37E-07** | **0.00045** | **-161** |
| *UGT2B7* | 5.83E-07 | 0.000465 | 37 |
| *APOBEC3H* | 6.86E-07 | 0.000475 | -41 |
| *HECTD1* | 6.43E-07 | 0.000475 | -36 |
| ***CTAG2*** | **6.82E-07** | **0.000475** | **-27** |
| *UGT1A5* | 7.16E-07 | 0.000475 | -41 |
| *DHX38* | 1.11E-06 | 0.000708 | -37 |
| *ZNF492* | 1.20E-06 | 0.000735 | 30 |
| ***IFNA16*** | **1.53E-06** | **0.000904** | **21** |
| ***MUC5AC*** | **1.73E-06** | **0.000985** | **147** |
| *LLGL2* | 3.12E-06 | 0.001706 | 38 |
| *NBPF3* | 3.21E-06 | 0.001706 | -52 |
| *XIRP2* | 3.64E-06 | 0.001871 | -51 |
| *PCDHB7* | 4.07E-06 | 0.002026 | 51 |
| *GZMH* | 4.92E-06 | 0.002305 | 22 |
| ***MUC21*** | **4.82E-06** | **0.002305** | **46** |
| *BTNL2* | 6.62E-06 | 0.003016 | 41 |
| ***FANCM*** | **9.68E-06** | **0.004059** | **-24** |
| ***PHLPP1*** | **9.22E-06** | **0.004059** | **-37** |
| *SELENOO* | 9.58E-06 | 0.004059 | 39 |
| *ACOX2* | 1.25E-05 | 0.00512 | -18 |
| ***HLA-DRB5*** | **1.32E-05** | **0.005254** | **177** |
| ***LAMB1*** | **1.61E-05** | **0.006221** | **-32** |
| *KCNK16* | 1.64E-05 | 0.006221 | 35 |
| ***PIGR*** | **1.73E-05** | **0.006354** | **23** |
| *IRGC* | 1.79E-05 | 0.006354 | -17 |
| ***SERPINB2*** | **1.83E-05** | **0.006354** | **22** |
| *CFAP47* | 1.85E-05 | 0.006354 | 33 |
| ***AR*** | **1.87E-05** | **0.006354** | **54** |
| ***LRIT2*** | **1.99E-05** | **0.006607** | **-34** |
| *KANSL1* | 2.06E-05 | 0.006696 | -37 |
| *PCDHB9* | 2.11E-05 | 0.006725 | 43 |
| *NBPF20* | 2.32E-05 | 0.00726 | -15 |
| *ADGRF1* | 2.42E-05 | 0.007379 | -17 |
| ***MDC1*** | **2.45E-05** | **0.007379** | **24** |
| ***SYCE1*** | **2.76E-05** | **0.007986** | **-18** |
| *INTS4* | 2.76E-05 | 0.007986 | -18 |
| *KRTAP1-1* | 2.98E-05 | 0.00849 | 18 |
| ***FCGBP*** | **3.07E-05** | **0.008577** | **-34** |
| *AMBN* | 3.28E-05 | 0.008893 | 14 |
| ***MKI67*** | **3.29E-05** | **0.008893** | **72** |
| *OR4A16* | 3.64E-05 | 0.009674 | -37 |
| *AGAP3* | 3.73E-05 | 0.009731 | -51 |
| *ZC3H13* | 4.19E-05 | 0.010316 | -22 |
| ***HGS*** | **4.21E-05** | **0.010316** | **-16** |
| *GOLGA6L10* | 4.27E-05 | 0.010316 | -61 |
| *PCDH8* | 4.08E-05 | 0.010316 | 14 |
| *LOC100134391* | 4.08E-05 | 0.010316 | -14 |
| *SOGA3* | 4.44E-05 | 0.010383 | 18 |
| *SOWAHB* | 4.45E-05 | 0.010383 | -26 |
| *KIAA1549* | 4.50E-05 | 0.010383 | -39 |
| ***RYR1*** | **4.86E-05** | **0.011064** | **60** |
| *RRP7A* | 5.03E-05 | 0.011298 | -20 |
| *ZDHHC8* | 5.14E-05 | 0.011372 | 17 |
| *KLC4* | 5.42E-05 | 0.011834 | -28 |
| *ZNF534* | 6.23E-05 | 0.013403 | -36 |
| *LRRC71* | 7.23E-05 | 0.015356 | 25 |
| *SPPL2C* | 7.96E-05 | 0.016635 | -41 |
| *SNX19* | 8.04E-05 | 0.016635 | -50 |
| ***ADRB2*** | **8.73E-05** | **0.017603** | **-31** |
| *MAST3* | 8.69E-05 | 0.017603 | -27 |
| *QRICH2* | 8.95E-05 | 0.017821 | -60 |
| *MAPT* | 9.90E-05 | 0.019472 | -32 |
| *HIST1H4H* | 0.000113 | 0.02165 | -21 |
| *MICAL2* | 0.000113 | 0.02165 | -27 |
| *TREML4* | 0.000125 | 0.023754 | -18 |
| ***SLC25A5*** | **0.000129** | **0.024139** | **-73** |
| ***ASPG*** | **0.000142** | **0.025365** | **-22** |
| *VWDE* | 0.000142 | 0.025365 | 54 |
| ***WDR90*** | **0.000139** | **0.025365** | **44** |
| ***CFHR1*** | **0.000137** | **0.025365** | **27** |
| ***ERVW-1*** | **0.000154** | **0.027196** | **-42** |
| *SPDYE16* | 0.000163 | 0.028563 | -17 |
| *PLEKHM1* | 0.000173 | 0.029974 | -22 |
| *PYROXD2* | 0.000194 | 0.033225 | 24 |
| ***CNDP2*** | **0.000214** | **0.035477** | **-18** |
| ***HLA-DRB1*** | **0.000216** | **0.035477** | **134** |
| *KIR2DS4* | 0.000215 | 0.035477 | -45 |
| *SLX4* | 0.00021 | 0.035477 | -27 |
| *TGM4* | 0.000228 | 0.037053 | 32 |
| *ZNF844* | 0.000231 | 0.037217 | 21 |
| ***APC*** | **0.000239** | **0.038039** | **-34** |
| *CPB2* | 0.000246 | 0.038791 | -29 |
| *CCDC137* | 0.000273 | 0.042667 | -27 |
| *DHDH* | 0.000296 | 0.045774 | 22 |
| *FAM114A1* | 0.000299 | 0.045786 | 27 |
| *OR4K15* | 0.000306 | 0.046483 | -31 |
| *CCDC180* | 0.000313 | 0.046696 | -34 |
| *UNC5D* | 0.000314 | 0.046696 | -16 |
| *NLRC4* | 0.000324 | 0.047775 | -16 |
| *RRP36* | 0.000333 | 0.048723 | 20 |
| *NXPE1* | 0.000337 | 0.048783 | -16 |
